# Microgreens: Functional Food for Nutrition and Dietary Diversification

**DOI:** 10.3390/plants14040526

**Published:** 2025-02-08

**Authors:** Tania Seth, Gyan Prakash Mishra, Arup Chattopadhyay, Partha Deb Roy, Mridula Devi, Ankita Sahu, Sukanta Kumar Sarangi, Chaitrali Shashank Mhatre, Yvonne Angel Lyngdoh, Visalakshi Chandra, Harsh Kumar Dikshit, Ramakrishnan Madhavan Nair

**Affiliations:** 1 ICAR-Central Institute for Women in Agriculture, Bhubaneswar 751 003, Odisha, India; mridula.devi@icar.gov.in (M.D.); ankita.sahu@icar.gov.in (A.S.); chaitrali.mhatre@icar.gov.in (C.S.M.); 2 ICAR-Indian Agricultural Research Institute, Pusa, New Delhi 110 012, Delhi, India; gyan.gene@gmail.com; 3 Bidhan Chandra Krishi Viswavidyalaya, Mohanpur 741 252, West Bengal, India; chattopadhyay.arup@bckv.edu.in; 4 ICAR-Indian Institute of Water Management, Bhubaneswar 751 023, Odisha, India; partha.roy@icar.gov.in; 5 ICAR-Central Potato Research Institute, Regional Station, Shillong 793 009, Meghalaya, India; yvonne.lyngdoh@icar.gov.in; 6 ICAR-Central Tuber Crops Research Institute, Thiruvananthapuram 695 017, Kerala, India; visalakshi.chandra@icar.gov.in; 7 World Vegetable Center, South Asia, ICRISAT Campus, Patancheru 502 324, Hyderabad, India; ramakrishnan.nair@worldveg.org

**Keywords:** microgreens, nutritional composition, growing conditions, sensory attributes

## Abstract

Microgreens are tender, edible seedlings harvested 7–21 days after germination containing a central stem, cotyledons, and true leaves. Known as a fresh, ready-to-eat functional food, they are mostly rich in vitamins, antioxidants, bioactive compounds, and minerals, with distinctive flavors, colors, and textures. These attributes make microgreens a valuable component in nutrition and health research. In countries like India, where low-income households spend 50–80% of their income on food, micronutrient deficiencies are common, particularly among women. Indian women, facing a double burden of malnutrition, experience both underweight (18.7%) and obesity (24.0%) issues, with 57% suffering from anemia. Women’s unique health requirements vary across life stages, from infancy to their elderly years, and they require diets rich in vitamins and minerals to ensure micronutrient adequacy. Microgreens, with their high nutrient density, hold promise in addressing these deficiencies. Fresh and processed microgreens based products can enhance food variety, nutritive value, and appeal. Rethinking agriculture and horticulture as tools to combat malnutrition and reduce the risk of non-communicable diseases (NCDs) is vital for achieving nutritional security and poverty reduction. This review compiles recent research on microgreens, focusing on their nutrient profiles, health benefits, suitable crops, substrates, seed density, growing methods, sensory characteristics, and applications as fresh and value-added products. It offers valuable insights into sustainable agriculture and the role of microgreens in enhancing human nutrition and health.

## 1. Introduction

Agriculture is key to achieving nutritional wellness, income generation, and sustainable livelihoods for millions. With growing consumer preferences for therapeutic, nutrient-dense, and low-calorie foods, new technologies are essential for producing these foods in greater quantities. Meanwhile, rapid urbanization and limited land access have increased the demand for low-calorie foods, like microgreens, among urban and peri-urban populations. Thus, farming methods that require minimal land, inputs, time, investment and yield nutrient-rich and profitable produce are needed for sustainable agriculture [1,2]. Microgreens cultivation, a smart agricultural practice using horizontal or vertical farming, can reduce the carbon footprint of food production, addressing both health and environmental issues [3]. These tender, edible seedlings harvested 7–21 days after germination offer unique flavors, textures, and nutritional value, earning them the nickname “vegetable confetti” [4,5,6]. Grown from various vegetable, herb, and grain seeds, microgreens can be grown year-round in small spaces, such as balconies or windows [7]. With high market value and minimal resource demands, microgreens present an economically viable enterprise for urban and rural communities [8,9]. This review covers the details of microgreens, including crop types, nutritional content, health benefits, cultivation systems, sensory attributes, and value-added products.

## 2. Historical Evolution of Microgreens

The historical roots of microgreens date back to the 1930s, when North American pharmacies commonly sold dried wheatgrass for medicinal use. By 1960s, sunflower, buckwheat, and radish were cultivated as winter “greens”, while the popularity of nutrient-rich “grasses” grew in the 1970s. During 1980s, the chefs in San Francisco began using microgreens from crops like arugula, basil, beets, kale, and cilantro for garnishing [10]. The term “microgreens” first appeared in the United States in 1998, and by the early 2000s, fresh microgreens became available in local North American retail outlets; by 2010, they were widely accessible in grocery stores [11]. The first scientific study on enhancing microgreens’ growth through seed treatments was published in the US [12]. By 2010, microgreens had gained popularity in research, with studies focusing on identifying suitable crops, media, and cultivation conditions. A bibliometric analysis from 2004 to 2023 reported that the US, Italy, and India contributed more to the field of microgreens research in terms of total publications, total citations, h-index, and citations per paper. These three countries were among the top ten most productive, accounting for around 65% of all published papers. The United States, Italy, and India have the most publications and the greatest influence on the issue of microgreens, with 133, 76, and 38 publications during the period under investigation, respectively [13]. In India, research on microgreens was minimal, and a study suggested that daily servings of fenugreek microgreens and mint leaves could be beneficial as dietary supplements [14]. Recently, a few studies have reported the importance of several microgreens, like lentil, mung bean, mustard, lettuce, red radish, pearl millet, and red cabbage, in India [6,15,16,17,18]. Now, research has shifted toward the effects of various light regimes on the yield and nutritional profiles of different microgreens. Since 2018, studies of value-added microgreens products have also emerged [19,20,21].

## 3. Market Trends for Microgreens

The global microgreens market can be categorized into four main segments: (i) green types, including Brassicaceae (e.g., cabbage, broccoli), Asteraceae (e.g., lettuce, chicory), Amaranthaceae (e.g., amaranth, spinach), Cucurbitaceae (e.g., cucumber, melons), Lamiaceae (e.g., basil, mint), and others, such as lentils, mung bean, and leeks; (ii) farm types, which consist of outdoor farming, greenhouse farming, and vertical farming; (iii) end uses, such as food and beverages and cosmetics; and (iv) regional markets, covering North America, Latin America, Europe, Asia Pacific, the Middle East, and Africa [22]. The United States is a significant contributor to the global microgreens market, followed by Canada and Mexico. In 2019, North America held nearly 50% of the global market share in terms of dollar sales, driven by large-scale microgreens farming and high restaurant consumption in the US [23].

Globally, the microgreens market is expected to grow at a compound annual growth rate (CAGR) of 7.5–8.0% from 2020 to 2025, reaching a value of USD 17.04 billion by 2025 [24]. In the US, the microgreens market is projected to grow at a CAGR of 10.1% during the same period [25]. Indoor farming, including hydroponic systems for microgreens, was valued at USD 25.40 billion in 2017 and rose to USD 40.25 billion by 2020 [22]. The retail price of microgreens is approximately five to eleven times higher than their cultivation cost [8], making commercial microgreens farming highly profitable. Growing multiple crop varieties together offers more flavor, color, and texture options, with “super mixes” being sold for between USD 66 and USD 110 per kilogram in the US [26]. In 2019, retail outlets like hypermarkets, supermarkets, and grocery stores accounted for 46.8% of the fresh microgreens market [27]. The retail sector is expected to grow at a CAGR of 11.4% from 2021 to 2028. Key trends in the microgreens industry include the adoption of advanced production technologies, the rise of indoor, vertical, and greenhouse farming, and the increasing demand for nutritious, high-quality food products [28].

In 2024, the microgreens market was valued at USD 245.77 billion, and it is projected to reach USD 335.16 billion by 2029, growing at a CAGR of 6.40% during the forecast period. The Asia Pacific region is expected to be the fastest-growing market, while North America remains the largest. Chefs are key drivers of the market, using microgreens to enhance both the flavor and presentation of dishes. Additionally, the cosmetics industry is contributing to market growth, with microgreens being processed into oils and ingredients for products like shampoos and skincare thanks to their high levels of vitamins A and B and various micro-elements. This makes them attractive to manufacturers in the personal care sector, further increasing demand. Although microgreens and herbs are among the most profitable crops, the limited varieties available for indoor farming may pose a challenge to market expansion. Despite the potential of hydroponics, its adoption in agriculture has been slower than expected. This could be due to the high cost of the hydroponics setup and also absences of assured microgreens markets [29]. Broccoli, lettuce, arugula, and basil are among the most widely cultivated microgreens grown using hydroponics and vertical farming methods. Demand for microgreens is increasing in developed countries, as they can be grown indoors year-round. With a growing population, microgreens offer a nutritious and sustainable option for diversifying the food supply, which is expected to drive market growth [30,31].

## 4. Popular Crops Used for Growing Microgreens

**Figure 1:**
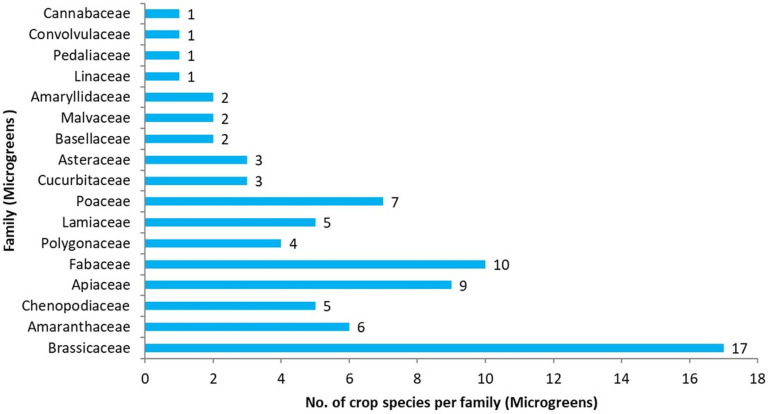
Contribution of different plant families in microgreens research.

**Figure 2:**
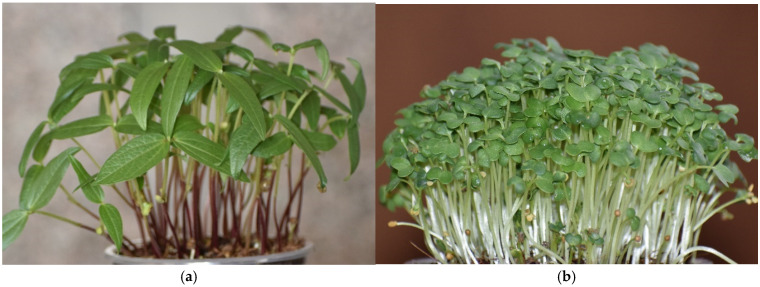
Microgreens of various species. (**a**) Mung bean microgreens. (**b**) Mustard microgreens.

A wide variety of crops, like legumes, cereals, pseudo-cereals, oilseeds, vegetables, and herbs, can be used to grow microgreens (Table 1; Figure 1 and Figure 2). Broccoli is the leading crop in the microgreens industry due to several health benefits [28]. Research findings show the nutritional supremacy of other microgreens, like finger millet, little millet, barnyard millet, pearl millet, mung bean, lentil, chickpea, flaxseed, and chia microgreens [7,15,17,32,33]. Among various groups, Brassicaceae families are widely used for microgreens production, followed by Fabaceae, Apiaceae, Poaceae, Amaranthaceae, Chenopodiaceae, and Lamiaceae [34]. The Solanaceae family of crops, like tomato, brinjal, and chilies, cannot be used for microgreens, as these contain anti-nutritional factors [35,36]. Microgreens offer an ideal carotenoid-rich food for space farming as a part of Space Life Support Systems (SLSSs) [37], as they require low photon flux and can be grown on synthetic media with little or no nutrient supplementation [38]. Microgreens are ideal candidates for astronauts’ functional salads [39] due to their requirement of low inputs and quick turn-around time when power, mass, and volume constraints prohibit the growth of crops to maturity. During spaceflight missions, more than 1.90 kg of edible biomass can be harvested annually from a 40 cm^2^ growth chamber by growing 26 crops of microgreens [40]. Around 1.00 kg of seeds is sufficient for 3-month production during a space mission, as 1.09 and 1.36 kg of fresh biomass can be produced from 6–7 g of seeds of cabbage and radish, respectively [41]. Therefore, 20 meals can be produced considering 50 g microgreens per meal.

## 5. Nutritional Importance

In the present busy lifestyle, people intake imbalanced diets and are consuming food much below the recommended levels, which results in various diet-related non-communicable diseases (NCDs), like underweight, obesity, diabetes, hypertension, cardiovascular diseases, cancer, etc. [47]. Urban households of low-income nations like India spend between 50 to 80% of their incomes on food [48,49] but still remain substantially food-deprived [50] and macro- and micro-nutrient deficiencies in the diet are common [51]. Current diets in most Indian states are lacking in 11 of the 25 essential nutrients [52], and approximately one-third of the Indian population follows a vegetarian or ovo-vegetarian diet [53]. Due to the reduced bioavailability of plant sources, vegetarian diets may lead to insufficient consumption of protein, vitamin B12, omega-3 fatty acids, and minerals, including iron and zinc [54,55]. In India, average vegetable consumption is 143g/male person/day and 138g/female person/day [56] against the RDA of 400 g/capita/day for normal health [57]. Rural women of Uttar Pradesh, Bihar, and Odisha states of India face a dietary gap, as their dietary diversity falls behind that of their other family members [51]. Indian women are also suffering from double burden of malnutrition (DBM), as 18.7% women between the age of 15 to 49 years are underweight while 24.0% are suffering from obesity and 57% women have anemia (NFHS-5) [58]. In order to prevent all types of malnutrition and lower the risk of NCDs, it is imperative that the agriculture and horticulture sectors be re-conceptualized as the key to reducing poverty, malnutrition, enabling substantial dietary shifts, and ensuring nutritional security. Women have the primary responsibility for ensuring the nutritional status of their family members, and by adding microgreens to their everyday meals, women can enhance household nutrition. Microgreens have the potential to solve these issues, as microgreens production requires little acreage and less input, time, effort, and money and provides nutrient-rich produce from farm to table [7]. Thus, microgreens farming can be a viable enterprise that can support the economic stability of both rural and urban women [9]. In addition, consumption of microgreens will also contribute to achieving the SDG for good health and well-being. Microgreens are considered fresh, ready-to-eat, nutraceutical-rich functional food [59,60,61], as they contain a variety of phyto-active substances that are beneficial to human health both on Earth and in space. Due to a few days of photosynthesis, microgreens are reported to contain much higher contents of bioactive compounds, phytonutrients, and antioxidants [62,63,64] (Table 2).

The nutrient contents of microgreens are recorded to be 30–40 times more than their mature counterparts [4,10,65]. Compared to sprouts, flaxseed microgreens showed higher chlorophyll, carotenoids, and phenol contents, while broccoli microgreens showed higher antioxidant contents [32,66]. Microgreens showed a wide range of vitamins and micro-nutrients, meeting 2–12%, 2–13%, 1–6% 28–116%, 28–332%, and 24–72% of the recommended daily intake of iron, zinc, selenium, vitamin C, vitamin E, and vitamin A, respectively [67]. Ascorbic acid content is potentially higher in the microgreens stage of plant development compared to sprouts, baby greens, and mature plants [66]. Mustard, basil, beet, red amaranth, peppercress, and radish microgreens contain 2.1–4.0 g of phylloquinone (vitamin K) per kilogram of microgreens [68]. Cilantro/coriander microgreens showed higher lutein/zeaxanthin content (10.1 mg/100 g FW), and the violaxanthin content (7.7 mg/100 g FW) compared to popcorn-derived microgreens is about 1.3 mg lutein/100 g FW and 0.9 mg violaxanthin/100 g FW [4].

Microgreens have great potential to diversify and enhance the human diet and address nutritional deficiencies due to their high content of phytochemicals [69,70,71]. They have anti-diabetic, anti-inflammatory, anti-cancer, anti-bacterial, anti-hypoxic, anti-hyperglycemic, anti-obesity, and anti-cholinergic properties [61,72,73,74,75]. Microgreens can potentially convert the food to a nutrient-dense recipe and help in the prevention of diet-related diseases [10] and high-altitude disorders of Indian troops [72]. Previous studies reported that 100 g of chicory and lettuce microgreens cultivated with a nutrient solution containing 58.4 or 117 mg K L^−1^ provides about 15.8–16.5% of the K daily intake recommended for patients affected by chronic kidney disease [76]. It is reported that microgreens do possess low levels of anti-nutrients, like nitrates [7,11,77,78,79], oxalates [7,67] and trypsin inhibitors (0.005 mg TIU/mg protein) [80], which is safe for consumption.

**Table 2:** Nutritional compositions of different microgreens.

Nutritional Components	Microgreens	References
Macro- and micro-minerals (Ca, Mg, K, Fe, Zn, Mn, Cu, Se, and Mo)	Lettuce	[11]
	Arugula, Broccoli, Brussel Sprouts, Chinese Cabbage, Cabbage Green, Cabbage Red, Cabbage Savoy, Cauliflower, Collard, Kale Chinese, Kale Red, Kale Tuscan, Kohlrabi Purple, Komatsuna Red, Mizuna, Mustard Dijon, Mustard Red, Pak Choi, Peppercress, Radish China Rose, Radish Daikon, Radish Red, Radish Ruby, Rapini, Rutabaga, Tatsoi, Turnip, Upland Cress, Wasabi, Watercress	[81]
	Kale	[82]
	Broccoli	[83,84]
	Basil, Swiss Chard, Rocket	[77]
	Chicory, Lettuce, Broccoli	[85]
	Bottle Gourd, Cucumber, Pumpkin, Amaranthus, Poi, Jute, Water Spinach, Radish, Palak	[65]
	Broccoli, Curly Kale, Red Mustard, Radish	[62]
	Coriander, Kohlrabi, Pak Choi, Cress, Komatsuna, Mibuna, Mustard, Radish, Tatsoi, Green and Purple Basil, Jute, Swiss Chard	[78,86]
	Lettuce	[79]
	Arugula, Broccoli, Red Cabbage, Red Beet, Red Amaranth, Pea	[63]
	Dill, Fenugreek, Red Amaranth, Green Amaranth, Spinach	[87]
	Celery	[88]
	Anise, Chervil, Caraway, Dill	[89]
	Black Gram, Mung bean, Chickpea	[64]
	Green Gram, Horse Gram, Mustard, Amaranthus, Ragi, Wheat	[7]
	Pea, Sunflower	[90]
Vitamins (C, K, E)	Arugula, Beet, Celery, China Rose Radish, Opal Radish, Green Daikon Radish, Cilantro/Coriander, Garnet Amaranth, Pea, Green Basil, Magenta Spinach, Mizuna, Opal Basil, Peppercress, Popcorn Shoots/Maize, Purple Kohlrabi, Purple Mustard, Red Mustard, Red Cabbage, Red Orach, Red Sorrel, Sorrel, Wasabi	[4]
	Bottle Gourd, Cucumber, Pumpkin, Amaranthus, Poi, Jute, Water Spinach, Radish, Palak	[65]
	Coriander, Kohlrabi, Pak Choi, Cress, Komatsuna, Mibuna, Mustard, Radish, Tatsoi, Green and Purple Basil, Jute, Swiss Chard	[78,86]
	Spinach, Carrot, Mustard, Radish, Roselle, Onion, Fenugreek, Sunflower, French Basil, Fennel	[67]
	Lettuce	[79]
	Broccoli	[84,91]
	Broccoli, Daikon, Mustard, Arugula, Watercress	[92]
	Alfalfa, Red Cabbage, Yellow Beet, Fennel	[93]
	Green Gram, Horse Gram, Mustard, Amaranthus, Ragi, Wheat	[7]
	Mung bean, Lentil, Red Radish, Pearl Millet, Mustard, Red Cabbage	[17]
Lutein	Basil, Parsley, Mustard, Kohlrabi, Mizuna	[94,95]
	Amaranth, Cress, Mizuna, Purslane	[96]
	Cabbage, Radish	[41]
Neoxanthin, violaxanthin	Cabbage, Radish	[41]
Caffeic acid and rosmarinic acid	Red Basil	[97]
Plant pigments (chlorophyll, carotenoids, anthocyanins)	Buckwheat	[98]
	Arugula, Beet, Celery, China Rose Radish, Opal Radish, Green Daikon Radish, Cilantro/Coriander, Garnet Amaranth, Pea, Green Basil, Magenta Spinach, Mizuna, Opal Basil, Peppercress, Popcorn Shoots/Maize, Purple Kohlrabi, Purple Mustard, Red Mustard, Red Cabbage, Red Orach, Red Sorrel, Sorrel, Wasabi	[4]
	Red Cabbage, Purple Kohlrabi, Red and Purple Mustard, Mizuna	[99,100]
	Chicory, Lettuce, Broccoli	[85]
	Wheat, Barley	[101]
	Coriander, Kohlrabi, Pak Choi, Cress, Komatsuna, Mibuna, Mustard, Radish, Tatsoi, Green and Purple Basil, Jute, Swiss Chard	[78,86]
	Spinach, Carrot, Mustard, Radish, Roselle, Onion, Fenugreek, Sunflower, French Basil, Fennel	[67]
	Lettuce	[79]
	Broccoli	[84,91,102]
	Broccoli, Daikon, Mustard, Arugula, Watercress	[92]
	Dill, Fenugreek, Red Amaranth, Green Amaranth, Spinach	[87]
	Green Basil, Red Basil, Rocket	[103]
	Flaxseed	[32]
	Celery	[88]
	Anise, Chervil, Caraway, Dill	[89]
	Kale, Swiss Chard, Arugula, Pak Choi	[104]
	Black Gram, Mung bean, Chickpea	[64]
	Alfalfa, Red Cabbage, Yellow Beet, Fennel	[93]
	Green Gram, Horse Gram, Mustard, Amaranthus, Ragi, Wheat	[7]
	Radish, Savoy Cabbage	[105]
	Pea, Sunflower	[90]
	Mung bean, Lentil, Red Radish, Pearl Millet, Mustard, Red Cabbage	[17]
	Flaxseed, Radish, Broccoli, Cabbage, Pak Choi, Beetroot, Red Amaranthus	[33]
Phytochemicals (polyphenols, flavonoids, antioxidant activity)	Buckwheat	[98]
	Red Cabbage, Purple Kohlrabi, Red and Purple Mustard, Mizuna	[99,100]
	Chicory, Lettuce, Broccoli	[99]
	Coriander, Kohlrabi, Pak Choi, Cress, Komatsuna, Mibuna, Mustard, Radish, Tatsoi, Green and Purple Basil, Jute, Swiss Chard	[78,86]
	Bottle Gourd, Cucumber, Pumpkin, Amaranthus, Poi, Jute, Water Spinach, Radish, Palak	[65]
	Finger Millet, Green peas, Sesame	[42]
	Soybean	[106]
	Chickpea, Mung bean	[15]
	Broccoli	[91]
	Beet, Amaranthus	[107]
	Broccoli, Daikon, Mustard, Arugula, Watercress	[92]
	Green Basil, Red Basil, Rocket	[103]
	Flaxseed	[32]
	Kale, Swiss Chard, Arugula, Pak Choi	[104]
	Anise, Chervil, Caraway, Dill	[89]
	Black Gram, Mung bean, Chickpea	[64]
	Alfalfa, Red Cabbage, Yellow Beet, Fennel	[93]
	Radish, Savoy Cabbage	[105]
	Mung bean, Lentil, Red Radish, Pearl Millet, Mustard, Red Cabbage	[17]
	Mustard	[108]
	Flaxseed, Radish, Broccoli, Cabbage, Pak Choi, Beetroot, Red Amaranthus	[33]

## 6. Growing Medium for Microgreens Cultivation

Successful microgreens production comprises aspects like selection of appropriate species, quality of seeds, seed density, substrates, and growing systems [35]. The growing media’s physical properties, including porosity (nearly 85%), water holding capacity (55–70% of total volume), and aeration (20–30% of total volume for roots), play an important role in the growth of microgreens [109]. Desired chemical properties of the media include optimum pH (5.5 to 6.5), electrical conductivity (below 500 μS/cm), free from heavy metals, and microbial contamination of *Salmonella* and *E. coli* [35,36]. Microgreens can be grown organically throughout the year on locally available inexpensive media like soil and soil-less media [110], and using modern agriculture techniques, like hydroponics, aquaponics [111,112], and through vertical farming [113] (Table 3). Compared to traditional farming systems, microgreens require 93–95% less production time and fertilizer and 158–236 times less water compared to mature parts to have equivalent nutrients [82].

Good-quality microgreens can be harvested from soil-based media [11,46,80,114] with minimum production cost. Among the soil-less substrates, cocopeat is a widely used growth medium for microgreens due to its low cost, easy availability, and bio-degradability [115]. Soil-grown fennel, mint, amaranth, and fenugreek microgreens contained higher nutritive value (calcium, iron, vitamin C, and beta-carotene) compared to cocopeat and water [116]. Chickpea microgreens, when grown in soil, exhibited the highest ash, total chlorophyll, minerals, flavonoid, phenolic content, and antioxidant activity, whereas cocopeat with nutrient-solution-grown mungbean microgreens contained high amino acids [117]. The performance of six microgreens (wheat, ragi, green gram, horse gram, amaranthus, mustard) in five media (sterile sand, cocopeat, coir mat, tissue paper, newspaper) under room conditions and rain shelter showed a high yield under rain shelter on cocopeat media, while newspaper-grown microgreens produced low biomass [7]. Sowing of small-seeded crops at a 0.50 cm depth and large-seeded crops at a 1.0–1.50 cm depth was found to be optimal [72].

A fusion of cocopeat, vermiculite, and perlite @ 2:1:1 was used for the production of bottle gourd, cucumber, poi, pumpkin, jute, Amaranthus, water spinach, radish, and palak microgreens [65], while cocopeat, vermiculite, and perlite (5:2:1) fetched the maximum yield in radish, cabbage, red cabbage, mustard, cauliflower, turnip, broccoli, knolkhol, garden cress, and coriander microgreens [72]. Use of locally available compost, like vermicompost alone [82] or in combination with cocopeat, other substrates [87,118], and spent mushroom substrate compost [104,119], were also found to be cost-effective. The radish microgreens when grown under artificial light using only water without any nutrient solution give 6–10 times more biomass 10 days after sowing [120]. The overall nutritional composition of the microgreens can be enhanced through fortification of growing media with certain micro-nutrients, while the content of some undesirable elements, such as Na and nitrates, can be reduced [35].

The growth of goldfish and arugula microgreens showed the equally effective nature of both sump filter systems (SFSs) and microgreens aquaponics systems (APSs) in maintaining water quality [111]. Generally, nutrient film technique (NFT) and deep flow technique (DFT) hydroponic systems are used for microgreens farming. The maximum fresh shoot weight (10.14 kg/m^2^) of table beet was achieved when seed balls were pre-germinated in moist vermiculite, followed by growing hydroponically using NFT and harvesting 15 days after planting [121]. The influence of selenium bio-fortification on the bioactive compounds and the antioxidant activity of wheat microgreens extract using the DFT hydroponic system was studied [122]. A hydroponic system with pine tree fibers for the production of mustard, radish, kale, and broccoli microgreens [62], a system with polyethylene terephthalate fiber pads for cauliflower and broccoli microgreens [123], and a system with growing pads for broccoli microgreens [84] were reported. An increase in the oxygen concentration in the nutrient solution promotes the growth of hydroponically grown lentil and wheat microgreens [112]. An excess nutrient solution flow rate of 6–8 L/min inhibits the root surface area and root growth in Swiss chard microgreens [124].

**Table 3:** Substrates used for microgreens production.

Substrates	Microgreens	References
Soil	Arugula, Beet, Celery, Opal Radish, Cilantro/Coriander, Garnet Amaranth, Pea, Green Basil, Magenta Spinach, Mizuna, Opal Basil, Peppercress, Popcorn Shoots/Maize, Purple Kohlrabi, Purple Mustard, Red Mustard, Red Cabbage, Red Orach, Red Sorrel, Sorrel, Wasabi	[4]
	Cabbage	[99]
	Cauliflower	[125]
	Chinese Spinach, Joseph’s Coat	[126]
	Lettuce	[11]
	Chinese Cabbage	[127]
	Wheat, Barley	[101]
	Red Amaranthus	[114]
	Tartary Buckwheat	[46]
	Barnyard Millet	[80]
Potting soil consisting of peat moss, vermiculite, perlite, and bark (4.5:1.5:1.5:2.5)	Buckwheat	[128]
Soil and cocopeat	Beetroot, Red Amaranthus, Flax, Cabbage, Broccoli, Pak Choi, Radish	[33]
	Mung bean, Adzuki Bean, Chickpea, Coriander, Fenugreek, Spinach, Mustard	[129]
Soil, water, and cocopeat	Fenugreek, Amaranth, Fennel, Spinach, Mint	[116]
	Black Gram, Mung bean, Chickpea	[117]
	Fenugreek, Mung bean, Cowpea, Horse Gram, Wheat, Sorghum	[130]
Water	Radish	[120]
Soil, sphagnum moss, cocopeat, vermiculite, perlite	Cabbage, Radish, Beetroot, Fenugreek, Palak, Basil, Green Gram, Peas, Lettuce, Wheat, Sunflower, Cucumber, Amaranthus	[131]
Sterile sand, cocopeat, coir mat, tissue paper, newspaper	Wheat, Ragi, Green Gram, Horse Gram, Amaranthus, Mustard	[7]
Coconut fiber and cellulose sponge	Radish, Savoy Cabbage	[105]
Commercial peat and perlite mix, coconut coir, spent mushroom compost, organic waste compost	Pea, Radish	[132]
Sphagnum peat	Kale	[133]
Hydroponic	Table Beet	[121]
	Green Daikon Radish, China Rose Radish	[4]
	Broccoli	[82,134]
	Radish	[135]
	Basil, Swiss Chard, Rocket	[77]
	Chicory, Lettuce	[76]
	Wheat	[122]
	Scallions, Basil, Cilantro	[136]
	Wheat, Lentil	[112]
Hydroponic with pine tree fibers	Mustard, Radish, Kale, Broccoli	[62]
Hydroponics system with polyethylene terephthalate fiber pads	Cauliflower, Broccoli	[123]
Hydroponics system with growing pads	Broccoli	[84]
Aquaponics	Arugula	[111]
Rockwool	Arugula	[137]
	Mizuna, Arugula, Green Basil, Cress, Radish	[138]
Vermiculite	Red Cabbage, Broccoli	[139]
Coir fiber	Arugula, Broccoli, Beet, Red Cabbage, Red Garnet Amaranth, Pea	[140]
Cocopeat	Pearl Millet	[21]
Commercially processed compost and coir dust (1:1/*v*:*v*)	Carrot, Kale, Fenugreek, Finger Millet, Green Peas, Green Radish, Lettuce, Mustard, Amaranthus, Sesame	[42]
Coconut coir, sand, rice husk ash (1:1:1)	Chia	[43]
Cocopeat and rice husk (1:1)	Carrot, Radish, Spinach	[141]
Cocopeat, vermiculite, and perlite (2:1:1 *w*/*w*)	Bottle Gourd, Cucumber, Poi, Pumpkin, Jute, Amaranthus, Water Spinach, Radish, Palak	[52]
Cocopeat, vermiculite, and perlite (5:2:1)	Radish White, Radish Pink, Cabbage, Red Cabbage, Mustard, Cauliflower, Turnip, Broccoli, Knolkhol, Garden Cress, Coriander	[72]
Coconut coir dust and vermiculite (3:1) and white sphagnum peat and vermiculite (3:1)	Radish	[142]
Coconut fiber, vermiculite, and jute	Green Basil, Red Basil, Rocket	[103]
Cocopeat, vermiculite, and sand (2:1:1)	Mung bean, Lentil	[16]
Cocopeat, vermiculite, and sand (1.5:1.5:1)	Mung bean, Lentil, Red Radish, Mustard, PearlMillet, Red Cabbage	[17]
Peat and perlite mix (70/30) and cellulose mat	Pea, Red Radish, Sunflower, Red Basil	[143]
Vermicompost	Broccoli	[82]
Cocopeat, vermicompost, and soil (9:2:1)	Dill, Fenugreek, Red Amaranth, Green Amaranth, Spinach	[87]
Cocopeat, vermicompost, and powdered leaves and fibers of *Synedrellanodiflora* (1:1:1)	Beet	[118]
100% spent oyster mushroom substrate layered with 2 mm of potting soil on top	Arugula, Basil, Catnip, Cilantro, Peppermint	[119]
Mixed media of vermicast, sawdust, perlite, and mushroom compost (3:2:2:3)	Kale, Swiss Chard, Arugula, Pak Choi	[104]
Agave fiber, capillary mat, cellulose sponge, coconut fiber, peat moss	Kohlrabi, Pak Choi, Coriander	[86]
Agro-industrial compost (54% vineyard pruning residues, 46% tomato residues, and 20% coffee as an additive) and peat @ 50:50	Mizuna, Pak Choi	[144]

## 7. Seed Priming and Seeding Density for Microgreens Cultivation

Microgreens can be cultivated regardless of the season; however, optimizing sowing density and the harvesting day will help in decreasing the production cost of microgreens [145]. The efficient growth of microgreens is mainly dependent on the seed quality, the mean seed weight, and the germination percentage for attaining maximum yield. Seed priming is a simple, eco-friendly, and cost-effective technique through which seeds are partially hydrated to the point where germination-related metabolic processes begin but the radicle has not emerged [146] and is generally used for enhancing seed germination, plant establishment, and stress tolerance. Matric priming of seeds (–1 MPa at 12 °C for 6 days in fine vermiculite) resulted in a 0.33–2.79-fold increase in shoot dry weight of the table beet and chard microgreens [12] compared to seed soaking in sodium hypochlorite, water, hydrochloric acid, and hydrogen peroxide. Agronomic bio-fortification of seeds through nutrient priming with zinc sulfate (ZnSO_4_) and zinc oxide nanoparticles (ZnO) @ 200 ppm resulted in higher bio-accessibility of the zinc in both pea and sunflower microgreens [90]. However, pre-germinating and priming seeds can reduce the production time and enhance the establishment rate and yield [12]. Basil microgreens raised from sodium selenate (Na_2_SeO_4_ @ 8 mg/L) enriched seeds have high selenium content and antioxidant capacity [147]. Bio-fortification with Na_2_SeO_4_ @ 10.0 mg/L in basil, cilantro, coriander, and scallion microgreens [136] and sodium selenite (Na_2_SeO_3_ @ 0.25–0.50 mg/L) in wheat microgreens [122] under a hydroponic system showed higher content of antioxidants, selenium, and other minerals. An estimated daily intake (EDI) of 92 mg/day, a nutrient contribution (NC) of 102.2%, and an average total vitamin C content of 108.2 mg/100 g FW were observed in broccoli microgreens bio-fortified with 0.5% ascorbic acid (AA) [84].

The seed sowing density of microgreens depends on the crop species, the substrate, and the growing conditions (Table 4) [118]. However, variation in the sowing density of the same crop was also reported in the sowing of arugula at 55 g seeds/m^2^ [137], while 75.6 g seeds/m^2^ [148] and 189 g seeds/m^2^ [138] resulted in the highest yield recovery. The seed density of 8 seeds/cell (109.81 g seeds/m^2^) produced a higher number of shoots and a maximum yield of radish microgreens [142]. A high seed rate was recommended for roselle, fenugreek, sunflower, fennel, mustard, and radish microgreens (300–450 g seeds/m^2^), while a lower seed rate (150–250 g seeds/m^2^) was recommended for carrot, spinach, French basil, and onion microgreens production [67]. In chia microgreens, 204 g seeds/m^2^ is reported as optimal for the highest yield [43], while for mung bean and lentil microgreens, 222.22 g seeds/m^2^ is reported as optimal for the highest yield [17]. A high correlation was found between the mean seed weight and the microgreens yield of mung bean (r^2^ = 0.73) and lentil (r^2^ = 0.78) genotypes [6]. Microgreens of wheat and ragi produce less biomass against a high seed density of 705 and 520 g seeds/m^2^, respectively, leading to a biomass to seed ratio < 1.0. Meanwhile, green gram, horse gram, and mustard microgreens required 850, 610, and 440 g seeds/m^2^, respectively, to obtain a higher biomass-to-seed ratio of 1.6–2.6 [7]. A sowing density of 1285 g seeds/m^2^ was found to be optimal for bold-seeded crop production, like green pea microgreens [148].

## 8. Growing Requirements for Microgreens

**Figure 3:**
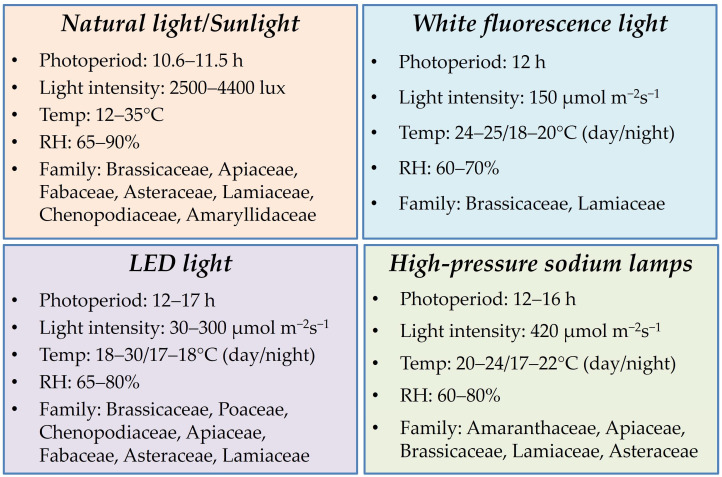
Light conditions (quality and quantity) requirement of various microgreens.

Microgreens can be grown under varied conditions, like open, inside-room conditions, greenhouses, and high altitudes either hydroponically or in soil or soil-less medium [7,72,150,151]. Successful microgreens production requires an average temperature between 20 and 30 °C and an RH of 50–80% depending on the crop species used (Table 5; Figure 3). Microgreens need a dark environment during the first three days of their germination; afterwards, they need to be exposed to adequate light for 12–16 h per day to promote growth [134,152].

Light is an important parameter that is directly involved in the microgreens’ morpho-physiology, yield, and nutritional composition [153,154]. Successful production of microgreens under natural light conditions was reported for various crop species, like broccoli, kale, radish, mustard, spinach, carrot, roselle, onion, fenugreek, sunflower, French basil, fennel, black gram, mung bean, and chickpea crops [62,64,66,67]. Chlorophyll absorbs mostly red (663 nm and 642 nm) and blue (430 nm and 453 nm) light, which are the primary light wavelengths [133]. The spectrum with the highest share of red light resulted in higher fresh and dry weights of 28 microgreens species [155]. Effects of different dosages of blue light in mustard, beet, and parsley microgreens showed 1.2 to 4.3 times higher concentrations of phytochemicals like carotenes, violaxanthin, zeaxanthin, lutein, chlorophyll, carotenoids, and tocopherols [156]. A red + blue (70R:30B) light spectrum, with photosynthetic photon flux density (PPFD) of 280 μmol m^−2^ s^−1^ and a 16 h photoperiod boosted the fresh yield, chlorophyll a, chlorophyll b, total carotenoids, anthocyanin, vitamin C contents, and total antioxidant capacity for red Amaranthus (*Amaranthus cruentus*) and leafy vegetable Amaranthus (*Amaranthus gangeticus*) microgreens [157]. Red, blue, or a mix of these lights was found to be more effective over white or yellow light alone in reducing the undesirable nitrates content in the microgreens [158,159].

Generally, use of artificial light, like light-emitting diode (LED) light [73,102,106,160], high-pressure sodium (HPS) lamps [104,161,162], and white fluorescent light [122,135,138], was preferred for growing of microgreens under a controlled environment. Low LED light intensity (120 ± 5.1 μmol m^−2^ s^−1^) enhanced a larger hypocotyl in cabbage, kale, mizuna, and mustard and also increased the cotyledon area in green and red mizuna compared to high LED light intensity (210 ± 5.9 μmol m^−2^ s^−1^) [163]. Significant increases in the hypocotyl length under blue LED light were reported in microgreens of mustard, kale, Amaranthus, and turnip greens [164,165]. The cultivation of beetroot, red Amaranthus, cabbage, flax, broccoli, Pak Choi, and radish microgreens in cocopeat in a growth chamber (20 ± 2 °C; 65 ± 2% RH; 55 μmol m^−2^ s^−1^ light intensity; 12/12 h, light/dark photoperiod) showed a higher nutritional value with desirable morphological traits [33]. Under bio-fortified conditions, the total antioxidant capacity (TAC) in radish microgreens increased nearly 1.7 times under bright light (4.6 ± 0.6 mg/g DW) over normal light (2.6 ± 0.7 mg/g DW), while in kale the increase was nearly 2.5times under bright light (9.2 ± 1.8 mg/g DW) over normal light (3.6 ± 0.5 mg/g DW). Similarly, radish microgreens were found to accumulate nearly nine times more total starch under bright light (191.9 ± 30.1 mg/g DW) over normal light conditions (20.9 ± 5.2 mg/g DW), whereas in kale the increase was nearly three times under bright light (106.2 ± 18.2 mg/g DW) over normal light (35.7 ± 15.4 mg/g DW) [166].

**Table 5:** Growing conditions, temperature, and light requirements for microgreens production.

Microgreens	Growing Conditions	Temperature and Relative Humidity	Light	References
Radish	Controlled growth chamber	25/18 °C (day/night)	White fluorescent light (150 µmol m^−2^ s^−1^, photoperiod 12 h)	[135]
Chicory, Lettuce, Broccoli	Controlled	20 °C RH: 85%	-	[85]
Arugula, Basil, Catnip, Cilantro, Peppermint, Spinach	-	RH: 75%	LED light (1000 lux) 12 h/day	[119]
Wheat, Barley	-	19 °C (7–27 °C) RH: 66% (29–100%)	-	[101]
Broccoli, Kale, Radish, Mustard	Unheated greenhouse	18 °C RH: 90%	Natural light	[62]
Bottle Gourd, Cucumber, Pumpkin, Amaranthus, Poi, Jute, Water Spinach, Palak, Radish	Greenhouse	27 ± 2 °C	-	[65]
Mizuna, Amaranth, Purslane, Cress	Growthchamber	22/18 ± 2 °C (day/night) RH: 65–75%	LED light combination red:green:blue (45:10:45%) (300 ± 10 µmol m^−2^ s^−1^; photoperiod 12 h)	[96]
Soybean	-	25 °C; RH:80%	LED light (photoperiod 12/12 h; intensity 30 μmol m^−2^ s^−1^)	[106]
Amaranth, Coriander, Cress, Green Basil, Purple Basil, Komatsuna, Mibuna, Mizuna, Pak Choi, Purslane, Swiss Chard, Tatsoi	Controlled walk-through climate chamber (phytotron)	24/18 ± 1 °C (day/night) RH: 60–70%	High-pressure sodium lamps (intensity 420 µmol m^−2^ s^−1^; photoperiod 12/12 h)	[161]
Spinach, Carrot, Mustard, Radish, Roselle, Onion, Fenugreek, Sunflower, French Basil, Fennel	-	25 ± 5 °C RH: 65 ± 10%	Sunlight (photoperiod 11.5 h; light intensity 2500–4400 lux)	[67]
Broccoli	Cold greenhouse	15.4 ± 5.8 °C	Natural light (4.6 to 9.2 MJ·m^−2^·d^−1^)	[66]
Wheat	Growth chamber by controlling light	25/20 °C, (day/night), RH: 60%	White fluorescence lamp (12 h light/dark; intensity 150 µmol m^−2^ s^−1^)	[122]
Kohlrabi Purple, Cabbage Red, Broccoli, Kale, Cabbage Green, Broccoli, Cauliflower, Kale, Turnip, Rutabaga, Brussel Sprouts, Mustard Red	Controlled growth chambers	RH: 70%	LED light combination red:green:blue (70:10:20)	[160]
Lettuce	Controlled growth chambers	24/18 ± 2 °C (day/night) RH: 70/80% ± 5%	LED light (12 h photoperiod; intensity 300 ± 15 μmol m^−2^ s^−1^)	[79]
Arugula, Broccoli, Beet, Red Cabbage, Red Garnet Amaranth, Pea	-	-	LED light (17 h photoperiod; intensity 62 μmol m^−2^ s^−1^)	[140]
Green Basil, Red Basil, Rocket	Controlled growth chambers	20 °C RH: 60–70%	LED light (intensity 65 µmol m^−2^ s^−1^; photoperiod 12/12 h)	[103]
Scallions, Basil, Cilantro	Growth chambers	19.7/18.7 °C (day/night) RH: 57.3%	Photoperiod of 16/8 h light/dark, PPFD 210 µmol m^−2^ s^−1^	[136]
Broccoli	Controlled growth chambers	22 ± 2 °C RH: 60 ± 5%	LED light (intensity of 50–70 μmol m^−2^ s^−1^) (12/12 h light/dark photoperiod)	[102]
Soybean, Pea, Rocket, Radish	-	18 °C	LED light (intensity of 270 μmol m^−2^ s^−1^) (16/8 h light/dark photoperiod)	[73]
Basil	Controlled growth chambers	21/17 ± 2 °C (day/night) RH: 65 ± 5%	HPS and LED light (15 h photoperiod)	[162]
Lettuce		20 ± 2 °C RH: 80 ± 5%	HPS and LED light (12 h photoperiod)	
Brussels Sprouts		24/18 ± 2 °C (day/night) RH: 70/80 ± 5%	HPS and LED light (12 h photoperiod)	
Tartary Buckwheat	Growth chamber	22 ± 1 °C	LED light (intensity of 50 μmol m^−2^ s^−1^) (16/8 h light/dark photoperiod)	[46]
Kale, Swiss Chard, Arugula Pak Choi	Greenhouse	24/22 °C (day/night) RH: 71%	600 W high pressure sodium lamp (16/8 h day/night)	[104]
Black Gram, Mung bean, Chickpea	Natural conditions	31–35 °C and 12–17 °C (max and Min) RH: 71%	Average sunshine and daylight of 7.8 h and 10.6 h	[64]
Mizuna, Arugula, Green Basil, Cress, Radish	Phytotron	24/18 ± 1 °C (day/night) RH: 60/70%	Fluorescent lamps 12/12 h photoperiod	[138]
Radish	Controlled growth chamber	20 ± 2 °C	16/8 h day/night	[120]
Kale	Controlled growth chamber	21/17 ± 2 °C (day/night) RH: 50–60%	LED light white, blue, and red (16/8 h day/night)	[133]
Radish, Savoy Cabbage	-	24/18 ± 2 °C (day/night) RH: 65 ± 5%.	LED light (400–700 nm and an intensity of 300 ± 10 μmol m^−2^ s^−1^); 12 h photoperiod	[105]
Mung bean, Lentil, Red Radish, PearlMillet, Mustard, Red Cabbage	-	20–30 °C RH: 65–75%	20 watts LED lamps (16/8 h light/dark)	[17]
Wheat, Ragi, Green Gram, Horse Gram, Amaranthus, Mustard	Room condition	29.1 °C RH: 73.23%	-	[7]
	Rain shelter	32.2 °C RH: 65.03%		
Cabbage, Radish	Growth chamber	24 ± 1 °C/18 ± 2 °C (day/night)	LED light (400–700 nm; intensity: 150–300 μmol m^−2^ s^−1^ PPFD); 12 h photoperiod	[46]
Pearl Millet	-	-	LED (8–10 h)	[21]
Mizuna, Pak Choi	Controlled growth chamber	23 °C RH: 75%	Blue/red LED light (14/10 h light/darkness)	[144]
Beetroot, Red Amaranthus, Cabbage, Flax, Broccoli, Pak Choi, Radish	Growing chamber	20 ± 2 °C RH: 65 ± 2%	Light intensity 55 μmol m^−2^ s^−1^; photoperiod 12/12 h (light/dark)	[33]

## 9. Harvesting Time of Various Microgreens Species

Harvesting of microgreens was performed when the cotyledons and true leaves were fully developed, usually from 7 to 21 days, as it differs from species to species (Table 6). Manual harvesting of seedlings above the substrate layer with the help of scissors, leaving the root portion, is more practical and cost-effective for small-scale production. Special attention should be taken to avoid sticking any growing media particles and seed integuments [35]. Mechanical harvesting is more feasible for a large-scale production unit as it reduces labour costs and increases production efficiency; however, studies reported that due to the plants’ small size and delicate nature, mechanical harvesting sometimes causes post-harvest damages, potentially affecting the shelf life and quality of microgreens [167]. Meanwhile, using Pepper Grinder harvesting techniques will allow for harvesting microgreens in microgravity without leaving debris in the spacecraft cabin [168]. Therefore, while mechanical harvesting offers potential benefits, its adoption depends on the scale of production and the ability to manage associated costs and quality control measures.

Due to perishability, the harvested microgreens are immediately washed and packed in polythene bags or clamshell boxes. The microgreens packed in a zip lock PPE bag can be stored for 9 days at 4 °C [7]. Harvesting of pea microgreens at different times, i.e., 5th [132], 7th [143], and 10th DAS [73,90,140], for obtaining a higher yield with desired quality was reported by different workers. Mung bean and lentil microgreens, when harvested 7 days after sowing (DAS), yielded a biomass ranging from 180 to 220 g/m^2^ and 140 to 160 g/m^2^, respectively [17]. Similarly, harvesting mung bean, mustard, and lentil microgreens at 7, 8, and 9 DAS, respectively, was identified as optimal [6,16]. For millet microgreens, pearl millet harvested at 10 DAS [17], barnyard millet at 12–14 DAS [80], and finger millet at 13 DAS [42] provided maximum yield. Late harvesting was recommended for aromatic herbs and spices, such as chervil, anise, dill, Chinese basil (perilla), green and purple basil (at 20 DAS), and caraway (at 23 DAS) [89,169]. However, dill harvested at 14 DAS was also considered optimal [87,170]. Additionally, harvesting at 20 DAS was reported to be optimal for arugula, broccoli, beet, red cabbage, red garnet amaranth [140], and peppercress [96] microgreens.

**Table 6:** Optimum harvesting time for different microgreens.

Microgreens	Harvesting (DAS)	Reference
Amaranthus (Green/Red), Arugula, Basil (Red), Beetroot, Broccoli, Cabbage (Chinese/Green), Carrot, Cauliflower, Chia, Cress, Cucumber, Fenugreek, Flax, Garden Cress, Indian Mustard, Jute, Kale, Kohlrabi, Lentil, Mizuna, Mung bean, Mustard, Pak Choi, Pea, Radish (White/Red), Roselle, Rutabaga, Spinach, Sunflower, Turnip, Water Spinach, Wheat	6–9	[6,16,17,33,43,65,67,72,82,90,101,132,137,138,141,143,160,171,172]
Amaranth, Barley, Barnyard Millet, Basella, Bottle Gourd, Broccoli, Brussels Sprouts, Cabbage (Red/Green), Carrot, Cauliflower, Fenugreek, Knolkhol, Mizuna, Mung bean, Mustard (Green/Red), Onion, Pak Choi, Palak, Pea, Pearl Millet, Pumpkin, Radish (Pink/White), Rocket, Safflower, Soybean, Spinach, Tartary Buckwheat, Turnip	10–12	[17,46,65,67,72,73,80,90,101,117,120,122,140,141,144,157,171,173,174]
Amaranth (green/red), Arugula, Cabbage, Dill, Fennel, Fenugreek, Finger Millet, Basil (French/Green), Kale, Lettuce, Mizuna, Mustard, Pak Choi, Radish, Sesame, Spinach, Swiss Chard, Table Beet	13–15	[42,67,87,104,105,121,138,163,170,172]
Coriander, Mizuna, Savoy Cabbage	16–18	[72,105]
Amaranth, Anise, Arugula, Beet, Broccoli, Caraway, Chervil, Chinese Basil/Perilla, Dill, Lettuce, Peppercress, Purslane, Red Cabbage	19–21	[79,89,96,140,169]

## 10. Sensory Attributes of Microgreens

The visual appearance, flavor, and texture of microgreens were the main determining factors for consumer acceptance, but the overall acceptability was correlated with the flavor score and the pH. The total phenolic contents were strongly correlated with flavor attributes like sourness, astringency, and bitterness [68]. On this account, red cabbage, sorrel, and peppercress microgreens received low consumer acceptability scores [4,68]. Meanwhile, Amaranthus, beet, coriander, and Swiss Chard microgreens have higher consumer acceptability due to their lower astringency, sourness, and bitterness traits [68,126,161]. Among the ten Thai local species used for a consumer satisfaction survey, leaf mustard microgreens received the highest score of 4.9/5.0, while hairy basil microgreens obtained a lower score of 3.7/5.0 [175]. Likewise, the average sensory score for all of the attributes of local farm broccoli microgreens ranged between 4.5 to 5.4/7.0 compared to commercial broccoli microgreens at 3.1–3.7/7.0 [91]. Mustard microgreens stored in 150 µm polythene bags at 5 °C for 14 days obtained high consumer acceptance 1–2.5/5.0 [108]. Consumer acceptability of beetroot and carrot microgreens received higher scores of 6.2/9.0 and 6.3/9.0, respectively [176].

The aroma profile of Chinese basil (perilla) was studied in two species, *Perilla frutescens* var. *frutescens* (green leaves) and *P. frutescens* var. *crispa* (red leaves). The *P. frutescens* var. *frutescens* produced a fruity, sweet, spicy, and herbaceous aroma, while *P. frutescens* var. *crispa* microgreens emitted a citrusy, spicy, and woody aroma [169]. Broccoli, red cabbage, and tendril pea microgreens showed the highest overall acceptability score over arugula, beet, and red Amaranthus microgreens [140]. Overall, it was found that knowledge of and familiarity with microgreens, sensory evaluation, and perceived benefits were the major indicators of consumers’ willingness to buy any microgreens species [140,151].

## 11. Food Safety of Microgreens

During germination, the seeds release a mix of carbohydrates and peptides, which attract a number of microbes present in the rhizosphere, thus making microgreens more prone to the microbial contamination than their mature counterparts [177]. A greater microbial load was generally found for sprouts over microgreens [178]. However, more microbial contamination has been recorded for hydroponically grown microgreens over soil- or growing-media-grown microgreens [5], which could be due to the constant warm temperature and humid conditions maintained for the hydroponic system. Although studies on the survival and growth of pathogens on microgreens are limited (Table 7), such studies are abundant for sprouts [60].

Microbial contamination can be easily overcome through the use of good agricultural practices, like use of uninfected seeds, seed treatment, use of clean utensils, and use of UV for the disinfection of hydroponic systems, etc. [5]. *Trichoderma harzianum* Rifai (strain KRL-AG2 G41) and *T. virens* (strain G-41) (ThTv) application to either the seed ball or to the growth media was found to be effective in reducing damping-off (*Pythium aphanidermatum* (Edson) Fitzp.) in beet microgreens 14 days after planting [185]. Safer microgreens can also be produced through the use of blue and UV wavelength lights, as these have anti-microbial properties [60,186,187,188]. As microgreens are very delicate in nature, it is almost impossible to eliminate microbial contamination using harsh sanitization treatment.

Although no foodborne outbreak associated with microgreens-consumption has been reported [189], they are still considered a vehicle for bacterial pathogens [135]. Because there are not enough data generated regarding the microbial safety of microgreens, more attention should be paid to studying the survival and proliferation of food-borne pathogens on microgreens grown and stored under different conditions and for different periods [36].

## 12. Food Applications of Microgreens

Microgreens have a very short shelf life, even under refrigerated (4 °C) conditions, and they can be kept fresh for 3–10 days at the most [190,191]. The nutritional properties of microgreens are affected during post-harvest handling. Therefore, microgreens are mostly consumed fresh just after harvesting. However, preparation of various value-added processed products using either fresh or dried microgreens powder not only increases the storage life but also enhances the nutritive and therapeutic values of the food. Microgreens can add value to regional cuisine due to their fresh, ready-to-eat functional and nutraceutical values. Microgreens are frequently used as garnishing ingredients because of their vibrant color, flavor, and aroma. They can be incorporated into breakfast and snack items like toppings in dosa, idli, roti, bread omelet, khichdi, and raita, and they can also be used as garnish in salads, soups, smoothies, sandwiches, pizzas, dhoklas, spaghetti, and noodles to enrich the nutrient content of the food [192]. There is also scope for adding microgreens in the preparation of various daily meals like curries and gravies.

Fresh microgreens of some crops, like mung bean and lentil, although rich in minerals and vitamins, still received lower acceptability among consumers due to their typical beany flavor. Processing of fresh microgreens into nutrient-rich drinks/juice helps in wider acceptability among consumers and increases market demand. Microgreens and fruit-based functional beverage blends containing fenugreek microgreens juice (20 mL), kinnow mandarin juice (40 mL), aloe vera juice (29.72 mL), sorbitol (8.5 mL), and stevia (1.78 g) per 100 mL had high protein, sodium, potassium, vitamin C, β-carotene content, phenols, and antioxidants [193]. Juice prepared from *Alternanthera sessilis* microgreens extract blended with ginger, sugar syrup, and salt had higher stability and sensory attributes [194]. An easy method to incorporate more healthy foods into our diets seems to be through energy drinks made with microgreens. Cold-pressed microgreens juices from broccoli, amaranth, and red beet are rich in bioactive compounds and can be characterized as novel functional products [195]. Microgreens farming technology has immense potential to be used to produce ingredients for energy drinks and food additives at a commercial scale. Thus, enhancing the quality of food using various microgreens based formulations appears to be an option to tackle the nutritional security of our population.

Mung bean microgreens extract powder (6%) and wheatgrass powder (6%) incorporated in rice muffins provided higher protein content, dietary fiber, phenolics, flavonoids, and antioxidant properties. Meanwhile, 2% mung bean microgreens extract powder incorporated into muffins gave higher scores for texture, flavor, appearance, overall acceptability, and aroma [117]. *Anethum graveolens* microgreens incorporated into pulse-based, gluten-free crackers gave high protein (28.4 g), dietary fiber (3.99 g), micronutrients, and antioxidant content and had low fat (9.19 g) per 100 g of crackers [170]. Conversion of microgreens into hummus containing chickpeas and microgreens in a 40:60 ratio gave a good amount of vitamin C, total carotenoids, iron, and calcium [21]. Wheat-based bread enriched with pea and lupin microgreens is also reported to enhance health-promoting secondary plant metabolites [19].

## 13. Conclusions

Microgreens have the potential to stand out as a notable functional food due to their densely packed nutrients, texture, flavor, and numerous significant health benefits. Microgreens production requires less input, time, effort, and investment and provides nutrient-dense produce and higher profitability compared to traditional farming. Short growing cycles, simple cultivation techniques, use of locally available, inexpensive media, and cultivation throughout the year promote environmental sustainability and gain popularity among health-conscious consumers. Consumer interest in microgreens, a new functional crop, is made easier when cultivating nutrient-dense microgreens in a variety of closed environments utilizing a range of soil and soilless technologies. There is little research being conducted on how to increase the safety and quality of microgreens through production and processing techniques. Microgreens are generally grown in indoor environments under high humidity, which sometimes attracts diseases and pest infestation. Therefore, proper quality control measures should be followed to ensure uniformity in flavor, texture, and nutritional content. Microgreens have a very short shelf life due to their delicate nature and high respiration rates. There are knowledge gaps regarding pre-harvest treatments, lighting, alternative post-harvest treatments, temperature management, growth substrates and production types, seed bio-fortification, and seed decontamination. Research about the scope of biofortified varieties of crops in microgreens production is not yet reported. Improving post-harvest handling techniques, including packaging, storage, and transportation, will help extend their shelf life and preserve their quality for consumers. Exploring new crops and bio-fortified varieties, creating new cultivars with improved nutritional profiles and flavors, increasing awareness and educating consumers about their nutritional value and culinary uses, and evaluating cutting-edge cultivation methods will stimulate market expansion and demand for sustainable microgreens production.

## Figures and Tables

**Table 1:** List of crops and plant families explored for microgreens.

Common Name	Scientific Name	Family	Microgreens Color
Radish	*Raphanus sativus*	Brassicaceae	Green/purplish green
Cabbage	*Brassica oleracea* var. *capitata*		
Knolkhol	*B. oleracea* var. *gongylodes*		
Mustard	*B. juncea*		
Broccoli	*B. oleracea* var. *italica*		
Chinese cabbage	*B. rapa* var. *pekinensis*		
Kale	*B. oleracea* var. *acephala*		
Cauliflower	*B. oleracea* var. *botrytis*		
Arugula	*Eruca sativa*		
Vegetable Amaranthus	*Amaranthus tricolor*	Amaranthaceae	Green/red
Red Amaranthus	*A. cruentus*		
Quinoa	*Chenopodium quinoa*		
Palak	*Beta vulgaris* var. *bengalensis*	Chenopodiaceae	Green
Spinach	*Spinacia oleracea*		
Beet	*B. vulgaris*		Reddish green
Swiss chard	*B. vulgaris* var. *vulgaris*		
Carrot	*Daucus carota*	Apiaceae	Yellow
Coriander	*Coriandrum sativum*		Green
Celery	*Apium graveolens*		
Parsley	*Petroselinum crispum*		
Fenugreek	*Trigonella foenumgraecum*	Fabaceae	Green
Pea	*Pisum sativum*		
Chickpea	*Cicer arietinum*		
Mungbean/green gram	*Vigna radiata*		
Horse gram	*Macrotyloma uniflorum*		
Lentil	*Lens culinaris*		
Alfalfa	*Medicago sativa*		Light green
Flaxseed	*Linum usitatissimum*	Linaceae	Green
Buckwheat	*Fagopyrum esculentum*	Polygonaceae	Green
Basil	*Ocimum basilicum*	Lamiaceae	Green/reddish green
Chia	*Salvia hispanica*		Green/reddish green
Maize	*Zea mays*	Poaceae	Yellow
Wheat	*Triticum aestivum*		Green
Pearl millet	*Cenchrus americanus/ Pennisetumglaucum*		
Finger millet	*Eleusine coracana*		
Little millet	*Panicum sumatrense*		
Barnyard millet	*Echinochloa esculenta*		
Onion	*Allium cepa*	Amaryllidaceae	Green
Bottle gourd	*Lagenaria siceraria*	Cucurbitaceae	Green
Cucumber	*Cucumis sativus*		
Pumpkin	*Cucurbita moschata*		
Sesame	*Sesamum indicum*	Pedaliaceae	Green
Sunflower	*Helianthus annuus*	Asteraceae	Green
Lettuce	*Lactuca sativa*		

Source: [4,8,17,32,42,43,44,45,46].

**Table 4:** Optimum seed rate for different microgreens, as reported by different researchers.

Microgreens	Seed Rate/Density Per m^2^	Reference
Arugula	~50–100 g/m^2^	[137]
Beetroot	~200–250 g/m^2^	[121]
Kale	~30,000–40,000 seeds/m^2^	[62,83]
Basil, Green Basil	~50–55 g/m^2^; ~190 g/m^2^	[77,138,148]
Swiss Chard	~240 g/m^2^	
Rocket	~45 g/m^2^	
Cilantro, Spinach	~30,000–46,000 seeds/m^2^	[119,136]
Chicory, Lettuce, Cabbage, Savoy Cabbage	~30,000–40,000 seeds/m^2^, ~80–100 g/m^2^	[17,33,85,105,131]
Cauliflower, Broccoli	~30,000–40,000 seeds/m^2^, 100–120 g/m^2^	[33,76,85,102,123,148]
Mustard, Radish	~30,000 seeds/m^2^, ~50–60 g/m^2^	[17,62,148]
Mizuna	~70,000 seeds/m^2^	[7,33,96,138]
Amaranth	~80,000 seeds/m^2^, ~50–100 g/m^2^	
Cress	~50,000 seeds/m^2^, ~190 g/m^2^	
Chia	~200 g/m^2^	[43]
Roselle, Sunflower	~350 g/m^2^	[67]
Carrot	~180 g/m^2^	
Fennel	~430 g/m^2^	
Onion	~240 g/m^2^	
Scallion	~3,4000 34,000 seeds/m^2^	[136]
White Knolkhol	~75 g/m^2^	[33,90,132,138,148]
Daikon Radish	~170–190 g/m^2^	
Pea	~1300 g/m^2^, ~10,000 seeds/m^2^	
Fenugreek, Wheat	~200 g/m^2^	[131]
Mung bean, Lentil	~30,000 seeds/m^2^	[6]
Chervil, Caraway, Dill	~60,000 seeds/m^2^	[89]
Anise	~70,000 seeds/m^2^	
Mung bean, Lentil	~220 g/m^2^	[17]
Pearl Millet	~140 g/m^2^	
Wheat	~700 g/m^2^	[7,33]
Ragi	~500 g/m^2^	
Green Gram	~850 g/m^2^	
Horse Gram	~620 g/m^2^	
Pak Choi	~80 g/m^2^	[33]
Flax	~200 g/m^2^	
Rapini	~50,000 seeds/m^2^	[149]

**Table 7:** Microbial contamination reported for some microgreens.

Microgreens	Microbial Contamination	Inoculation/Storage	Reference
Radish	*E. coli* (O157:H7 & O104:H4)	-	[178]
Eight different species	Shiga-toxin-producing *E. coli*	Inoculated under hydroponics	[179]
Swiss Chard	*Salmonella enterica*	Contaminated water irrigation	[180]
Radish	*E. coli* (O157:H7)	Soil substitute and hydroponics	[135]
Rapini	Lower microbial populations	Recycled fiber mats	[181]
Radish	*Listeria monocytogenes*	Soil substitute and hydroponics	[182]
Kale, Mustard	Murine norovirus (MNV)	Hydroponics	[183]
Arugula, Kale, Lettuce, Mizuna	*E. coli* (O157:H7)	Stored in a refrigerator	[184]
